# Programable and Spatially Conforming Assembly of Engineered Living Materials Onto Electrodes via Redox

**DOI:** 10.1002/smsc.70331

**Published:** 2026-07-12

**Authors:** Chen‐Yu Chen, Monica J. Chu, Fauziah Rahma Zakaria, Eunkyoung Kim, Divya Muthusamy, Gregory F. Payne, William E. Bentley

**Affiliations:** ^1^ Fischell Department of Bioengineering University of Maryland College Park Maryland USA; ^2^ Institute for Bioscience and Biotechnology Research University of Maryland College Park Maryland USA; ^3^ Robert E. Fischell Institute for Biomedical Devices University of Maryland College Park Maryland USA

**Keywords:** bioelectronics, electrobiofabrication, electrochemical sensing, hydrogel

## Abstract

We developed an electrobiofabrication methodology that assembles well‐defined cell/gel formations directly onto electrodes. For this, we oxidatively crosslinked terminal thiols of a 4‐arm thiolated polyethylene glycol (PEG) by the purposeful addition of a ferrocene redox mediator to a PEG/cell assembly solution and the application of an oxidizing charge to an electrode. Because the resulting disulfide bonds are created near the electrode, the crosslinked hydrogel assembly is defined by the electrode dimensions and the time over which the oxidative potential is applied. Results indicate a strong positive correlation between the mediator concentration, the delivered oxidative charge, the number density of cells in the assembly solution and the subsequent gel thickness and density. In all cases tested, the viability of the assembled cells (*E. coli* bacteria) was near 100%. We further demonstrated a gravity‐mediated layering methodology to create spatially defined interfaces, as well as electroassembly onto various conductive materials of nearly arbitrary shape. These results represent a means for electronic or “programed” assembly of cell laden hydrogels, enabling further study of cell–cell interactions, cell‐device interactions, biosensing, device  ⇔ bio communication, and several applications such as electrogenetics wherein cell genetic circuits are actuated by application of electrical potentials using a redox‐enabled communication modality.

## Graphical abstract

We are developing electronic biofabrication methodologies for the assembly of viable, functioning cells onto conductive surfaces of nearly arbitrary shape. To do this, we electrochemically crosslink thiolated polyethylene glycol hydrogels, capturing the cells within the gels. No mechanical devices are needed; rather, one applies an oxidizing potential to an electrode or other conductive surface. It even works for pencil lead!

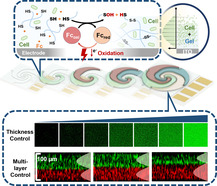

## 1 Introduction

Over the last few decades, semiconductor technologies transformed our abilities to access, store, process, and communicate information by enabling increasingly smaller, cheaper, more powerful, interconnected, and easier‐to‐use electronic devices; the Internet of Things (IoT) being a cutting‐edge expression of this transformation [1]. We suggest that synthetic biology may enable the extension of these abilities to include biological and living systems—by “rewiring” and “programing” cellular processes that manipulate chemical and biological information at the molecular scale [2, 3]. The addition of biological elements brings features like biocompatibility, low energy consumption, and evolutionary adaptability into the fold [4]. Indeed, devices that freely exchange information between the electronic and biological worlds would represent a completely new societal paradigm (e.g., as anticipated by the Internet of Bio‐Nano Things [5]). Application areas projected to undergo radical transformation include: human health, sustainability, energy, security, and food. While this represents an exciting vision, technical advances in this direction have been slow and have not kept pace with mainstream semiconductor technologies—we believe for two principal reasons: i) the physical integration of labile biological components into functional electronic systems is technically challenging [6], and ii) there is no clear and unified technology to establish cost effective, dimensionally feasible, rapid, reliable, and scalable communications between the electrical and the biological domains. In this work, we address the first of these challenges by significantly expanding an electrobiofabrication methodology we previously developed for the assembly of cells and hydrogels onto electronics platforms [7]. This report thus furthers information exchange in that the assembly of these “engineered living materials (ELM)” is electronically controlled via the redox‐enabled communication modality common to both. That is, redox‐active compounds are a medium for information exchange between biological systems and electrodes [8, 9, 10].

**FIGURE 1:**
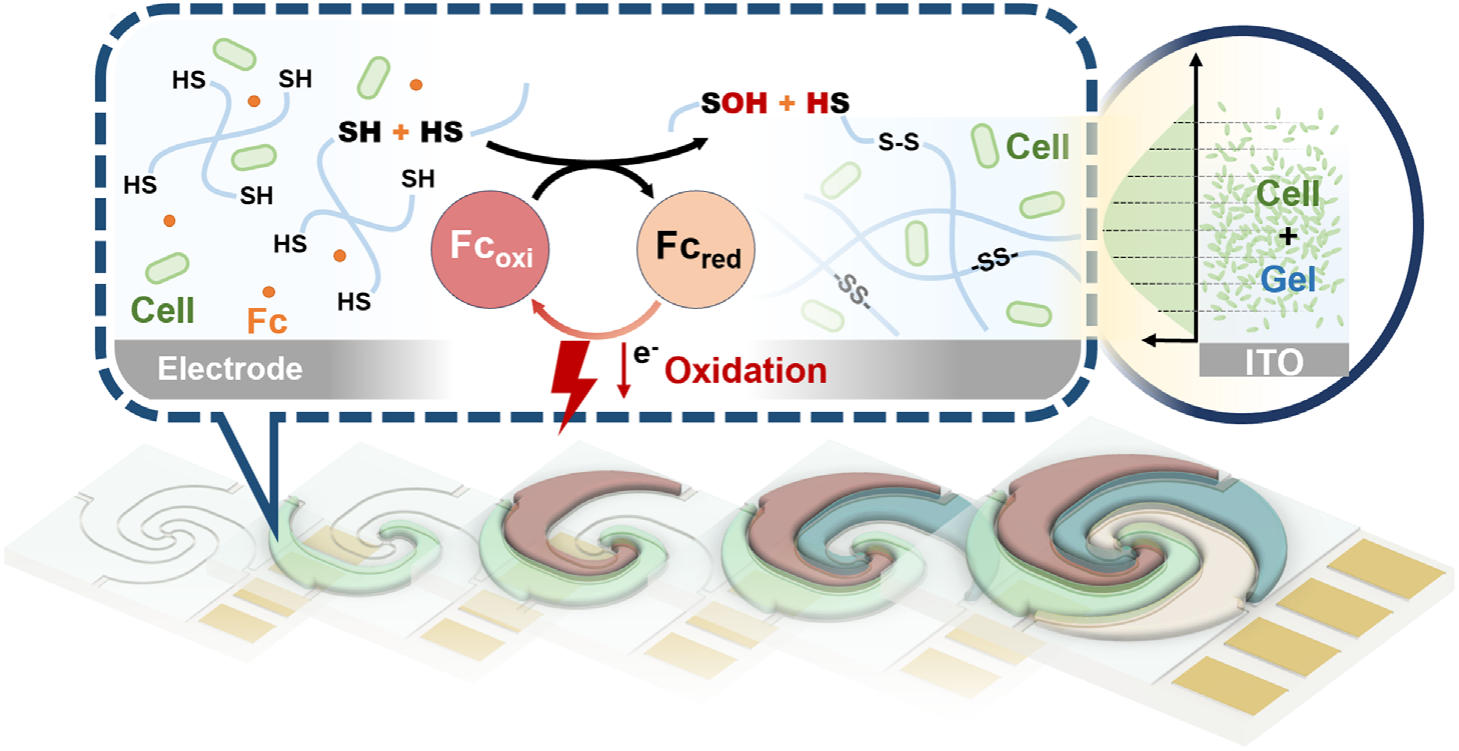
Electrobiofabrication for cell assembly in spatially defined hydrogels. Electroassembly is enabled by oxidative crosslinking of thiolated polyethylene glycol (PEG) monomers. Ferrocene (Fc) is oxidized at an electrode, it then oxidizes PEG‐thiols creating disulfide bonds that entrap cells in the growing hydrogel. Electrodes of various shapes and conductivities enable spatially resolved assembly (note lollipop electrode structures). Factors influencing the assembly process such as, potential gating and dynamic application, mediator and cell concentrations, enable designer assembly of these “living” materials that in turn, retain function of the assembled cells.

By applying redox‐enabling electronic signals to stimulus‐responsive biopolymer solutions such as chitosan or alginate [11, 12, 13, 14], or to synthetic systems designed for redox‐triggered crosslinking [15], one can electronically “program” assembly directly onto electrodes. Neither mechanical devices or interventions, nor human interactions are needed, just an applied potential [15]. In this report, we supplemented a solution containing 4‐arm thiolated polyethylene glycol (PEG) with the redox mediator 1,1′‐ferrocenedimethanol (Fc) so that an applied oxidizing potential to an immersed electrode facilitates the formation of polyethylene glycol (PEG) hydrogels via catalyzed disulfide bond formation between the thiolated‐PEG monomers (see Figure 1) [16, 17]. Using a convenient 3D‐printed device that enables optical and electrical I/O functionality [10, 18], we enable the rapid and reproducible assembly of PEG hydrogels directly onto the electrode, with boundaries defined by the electrode geometry. When cells are suspended within the PEG precursor solution, they are incorporated into the forming gel, enabling cell–hydrogel composites [19]. Importantly, this approach preserves both cell viability and cellular function following encapsulation [20, 21]. We fully explore effects of mediator and cell concentrations, applied charge, and even segmented depositions to enhance our understanding of the simplicity and flexibility of our approach.

In results, here, we incorporated engineered bacteria that express an optically quantified green fluorescent protein as an indicator of biological function and cell viability. So, by engineering advanced functions into the biological molecules and/or cells that are fixed or captured within these matrices, the resulting engineered living materials (ELMs) can provide highly intricate modalities for communication [21, 22, 23]. That is, beyond their noted potential as smart probiotics that treat human diseases [24] to living matrices that restore the environment [25], ELMs offer tremendous potential for creating bidirectional information exchange between biological systems and electronic devices [8, 19]. For example, cells engineered to secrete redox‐active phenazines can support cell‐to‐electrode communication when the electrode potential drives mediator oxidation/reduction [26, 27]. Conversely, cells engineered with oxidative stress‐related genetic circuits can be immobilized on electrodes so that electrode‐generated redox activities (e.g., H_2_O_2_ or oxidized phenolic, acetosyringone [28, 29, 30, 31]) actuate gene expression in situ, enabling electrode‐to‐biological signaling. Building on these capabilities, redox‐responsive eCRISPR modules have been demonstrated that connect electronic inputs to programable, multiplexed control of intracellular networks as well as population‐level responses [21, 22]. While the living components of the assembled hydrogels confer interesting and enabling functionalities, we have not fully explored their assembly. This is the focus of the present report.

## 2 Results

### 2.1 Cell Entrapment in PEG‐Based Hydrogel Assemblies

**FIGURE 2:**
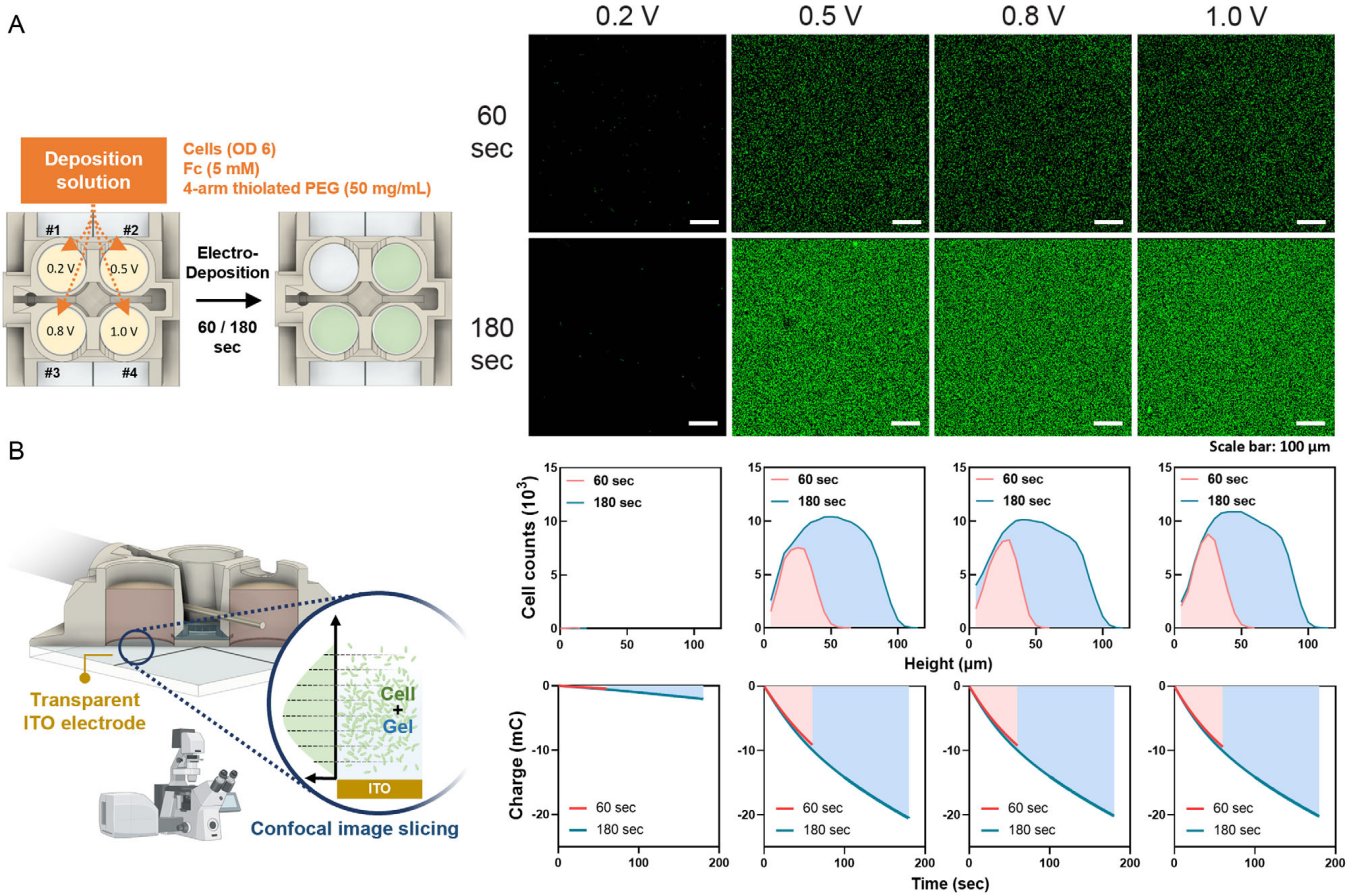
Electrobiofabrication for cell/PEG hydrogel assembly onto ITO. Fluorescence (top‐view) images acquired by confocal microscopy enable visualization of entrapped cells in the gels. (A) Several oxidative potentials (0.2, 0.5, 0.8, and 1.0 V) were tested for cell entrapment within 4‐well 3D printed optoelectrochemical device [10]. When the oxidative potential was low (i.e., 0.2 V) there was neither robust gel formation, nor cell deposition. The results indicate that oxidative potentials above 0.5 V provide similar cell entrapment profiles. Fluorescence images show uniform densities as a function of vertical distance irrespective of applied potential. (B) (left) Schematic shows a cutout view of the deposition suspension in wells and resultant vertical profile. (right) Cell counts per 5 µm slices acquired using confocal microscopy also revealed that the numbers and distributions of trapped cells in the PEG gel were strongly correlated with the time over which charge was applied to the ITO electrodes. The rates of applied charge (integrated current over time) were also uniform across applied potentials; resultant cell /gel materials were also similar, seemingly irrespective of potential (note that the characteristic Nernst potential of Fc for these materials is ≈+0.25 V, thus all potentials above this point should exhibit similar oxidation profiles).

We first explored the correlation between oxidative potential and cell/gel morphology (gel thickness and cell distribution). We suspended *E. coli* reporter cells (@OD_600_ = 6) engineered to constitutively express GFP (DH5α‐sfGFP) in a deposition solution (50 mg/mL thiolated‐PEG, 5 mM Fc) and applied a range of oxidative potentials (0.2, 0.5, 0.8, and 1.0 V) for different times (60 and 180 sec), triggering the disulfide crosslinking [10, 17]. As shown by the representative composite fluorescence images (Figure 2A), neither captured cells nor hydrogel were detected using an oxidative potential of 0.2 V irrespective of the duration. When the oxidative potential was raised to 0.5, 0.8, and 1.0 V, we found strong fluorescence from the cells entrapped within assembled PEG networks. We also noticed that the density and distribution of cells in the 2D (i.e., horizontal) plane appeared to be consistent across the applied potentials and their durations. In results not shown, after examining five subfields for each image we confirmed that the distribution across the electrode was highly uniform. In previous work, these methodologies, resulted in assembled hydrogels of Young's modulus of ≈0.4 kPa; we had further found that small molecules could freely diffuse into and out of these gels, while large proteins were retained once assembled [17, 19, 32]. Here, using confocal microscopy, we evaluated gel thickness and vertical cell distribution (Figure 2B, left panel). Using Z‐stacked images, we found the vertical distribution of cells was bell‐like in shape; the peak was consistently at the middle of the PEG gel, irrespective of the deposition time. Accordingly, the charge accumulation profiles (Figure 2B, right panel) were virtually identical, suggesting that rather than the applied potential, the rate‐determining steps for gelation were associated with charge, including the transport of Fc and/or its reaction kinetics with thiolated PEG. For convenience and to ensure robust gel formation, 0.8 V was chosen for later tests. Overall, this methodology provided a simple electrochemical methodology for the assembly of this living material onto an optically clear ITO electrode.

### 2.2 Controlled Cell Distribution

**FIGURE 3:**
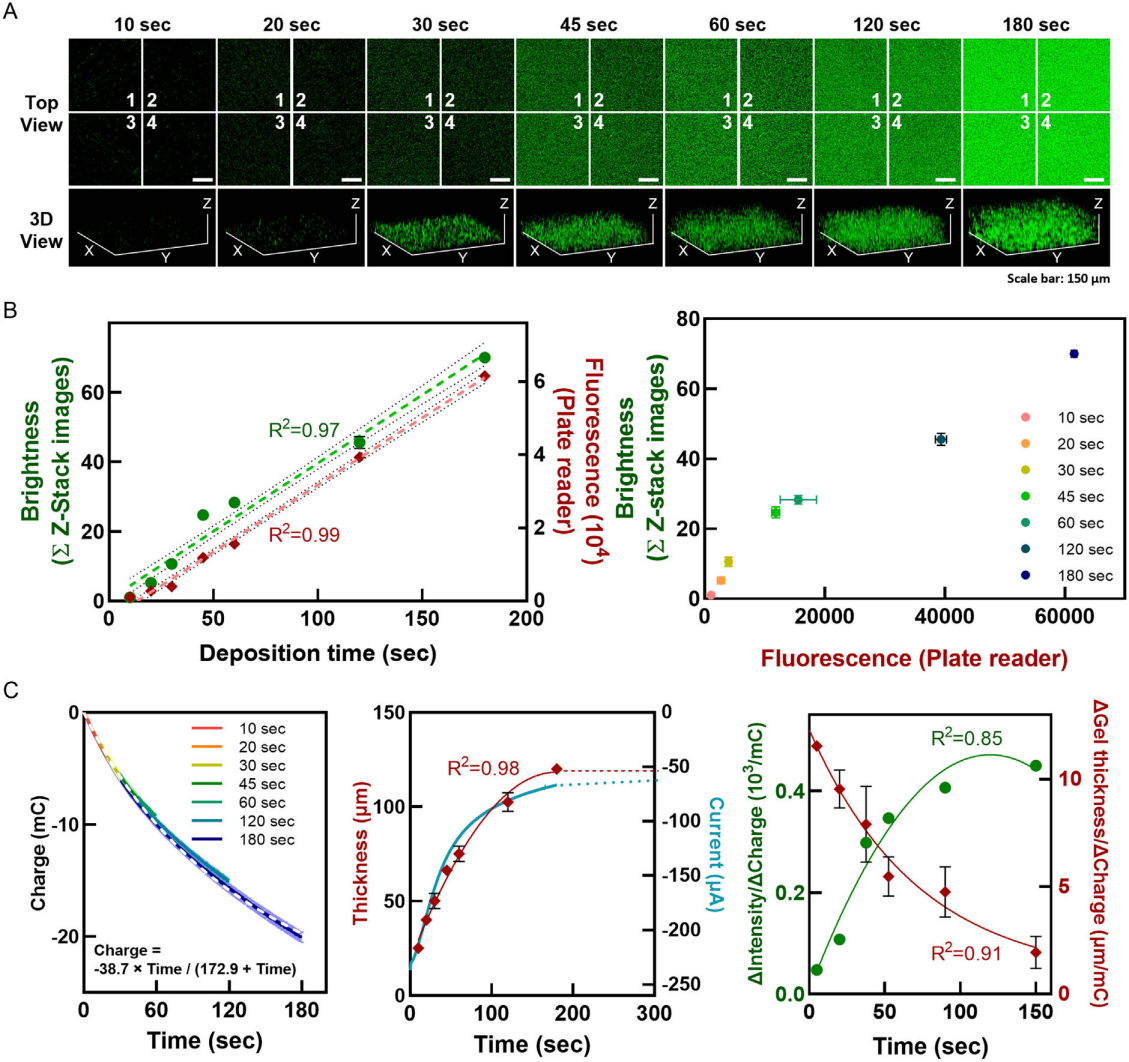
Cell uniformity in the assembled PEG gel. (A) All 4 wells on the device were tested using the same cell deposition solution (OD_600_ = 6, 5 mM Fc, and 50 mg/mL thiolated‐PEG) and oxidative potential (0.8 V) over different deposition periods (from 10s to 180 s). The fluorescence increased consistently with time. Variability within a well and between wells (indicated by white numbers) was small. (B) Results from image processing (“brightness”) and plate reader measurements (“fluorescence”) showed a linear increase with time. Moreover, a near linear relationship was obtained between measurements. (C) The charge profiles for different durations were nearly identical (left); the currents and measured thicknesses were fit with a hyperbolic model with high correlation coefficient (middle). Gel thickness and fluorescence intensity when normalized by charge showed rapid gelation at early times and with high levels of redox reaction; these slowed over time suggesting decreased crosslinking at later times and at higher vertical position. *Data are presented as mean ± SD (*n* = 4).

We next sought to more fully characterize the dynamics and reproducibility of cell/gel assembly onto ITO electrodes using +0.8 V (Figure 3). Using the specially constructed microwell sensing and actuating device shown in Figure 2 [10], we loaded 100 µL of deposition solutions containing DH5α‐sfGFP cells (OD_600_ = 6), thiolated‐PEG (50 mg/mL), and the redox mediator, Fc (5 mM), into each of 4 electrode‐bearing wells. We then followed the previously established protocol and applied +0.8 V to the ITO electrodes for several time periods (10–180 s). Figure 3A depicts the complete assembly of Z‐stack images yielding the overall fluorescence over the indicated deposition times. We also quantified fluorescence using the plate reader's fluorescence detection. As illustrated in the left panel of Figure 3B, the cell fluorescence intensities varied linearly with the deposition time (R^2^ values of 0.97 and 0.99, respectively). Not surprisingly, we found a strong correlation between both optical measures (right panel of Figure 3B). For convenience, in further tests, we report cell assembly using measurements from the plate reader unless otherwise indicated. Importantly, the relative standard deviation (RSD) of cell fluorescence measured from the plate reader was calculated across all tested deposition durations (10, 20, 30, 45, 60, 120, and 180 s), yielding values of 2.97%, 16.51%, 11.16%, 5.66%, 11.94%, 2.39%, and 0.77%, respectively. The fluorescence output was strongly related to the applied charge. Results depicted in the left panel of Figure 3C show that the charge profiles of all 28 wells followed the same trajectories (an R^2^ value of 0.99 when fitted with a hyperbolic model). Together, our findings demonstrate a high degree of reproducibility of the cell/gel assembly using the ITO device. That is, the applied charge, gel thickness, cell fluorescence within a gel and between gels, were all quite consistent.

We next attempted to tease out the assembly dynamics in even more detail. As shown in Figure 3C (middle panel), the gel thickness increased rapidly at first and then slowed over time. In Figure S1, we show how the decreased rate of thickness growth varied as the square root of time α $t^{\frac{1}{2}}$ (R^2^ = 0.98), potentially consistent with cells settling in the cases where cells interfere with gelation (see Supporting Information). In Figure 3C (right panel), we plotted the change in cell assembly (measured by direct spectrophotometer fluorescence) divided by the change in charge over the entire course of deposition and for all tests. Interestingly, initially the cell deposition per unit charge closest to the ITO surface was low, and this increased with time as the gel was formed upward. This suggests that the applied charge, which is due to the energy of crosslinking, is higher near the surface. Correspondingly, there were fewer cells entrapped nearest the probe. As the cell number increased towards the middle of the layer, the amount of charge associated with gel formation decreased. Then, as the gel became even thicker, the number of entities that would accept the applied charge (i.e., the oxidized Fc) was reduced and there was increasingly less energy associated with crosslinking, less gel formation, and ultimately a maximum height was achieved. This may reflect hindered mediator diffusion or reaction within the growing gel layer [33, 34] (Figure S2). We further evaluated the structural integrity and cell retention of the electroassembled ELMs by carrying out leaching and proliferation assays. Following a triple‐wash protocol and subsequent 3‐hour incubation in PBS, we found the hydrogels remained stable and with no visible alterations in cell morphology or gel properties. We then transferred gels to LB media for 8 h at 37°C; the entrapped cells proliferated into distinct, localized micro‐colonies within the PEG lattice, while negligible planktonic growth was detected in the surrounding media. These results indicate that the redox‐induced matrix provides robust physical sequestration while maintaining cell viability (Figure S3).

**FIGURE 4:**
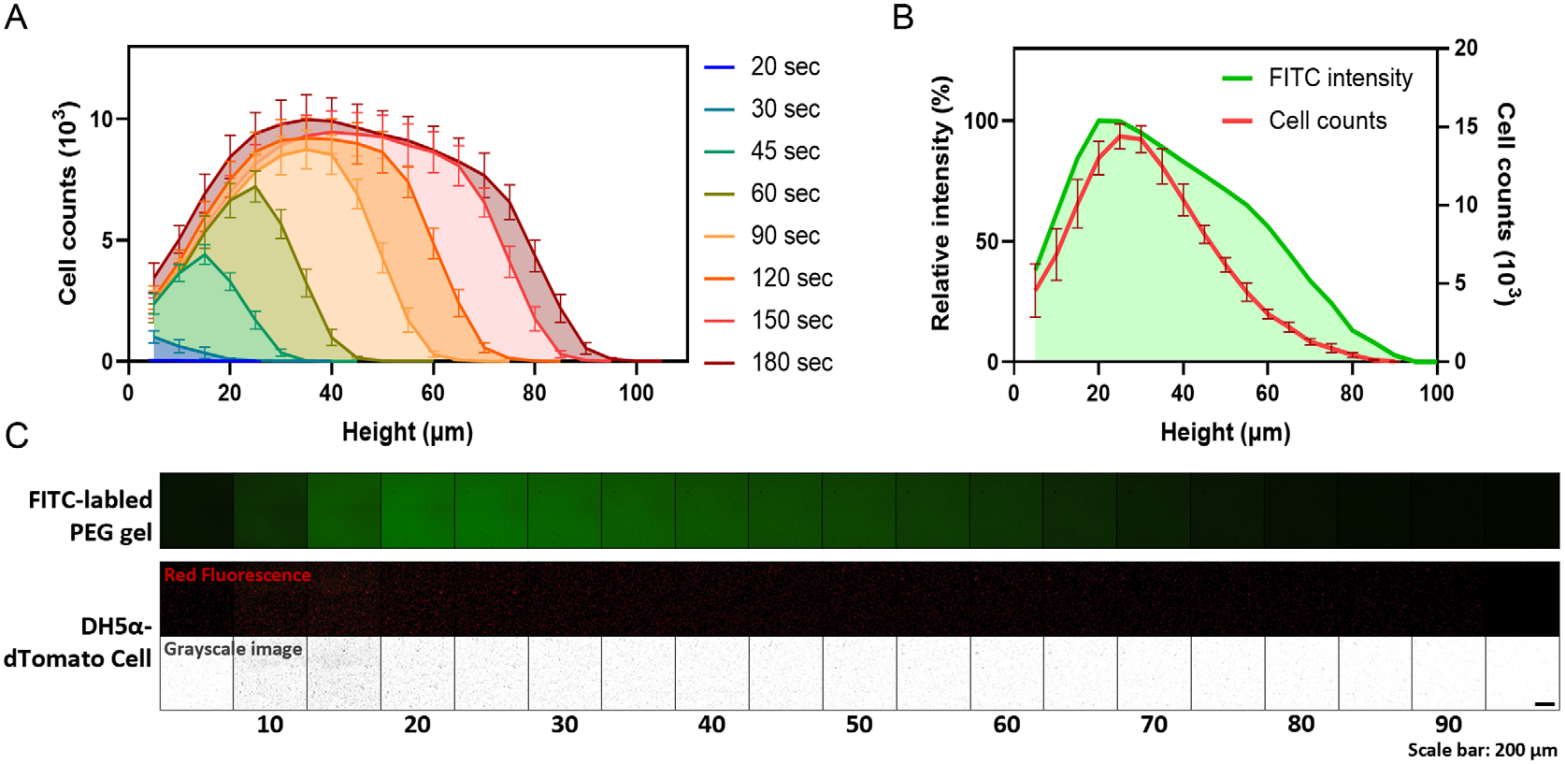
Cell distribution in electroassembled hydrogels. (A) Gelation and cell entrapment progress vertically in time, indicated by the gel height (*X*‐axis) and cell density (*Y*‐axis). At early times (20, 30, 45, 60 s), the cell number in the hydrogel grows substantially at any given height. As time progressed, newly entrapped cells appeared at greater distances from the electrode and few cells were added at lower heights. Thus, when an apparent maximum in cell density was reached, the growth in the number of entrapped cells was mainly contributed to by the increase in gel thickness. (B) Fluorescently labeled thiolated‐PEG monomers indicate crosslinking. The fluorescence profile quantified from confocal Z‐stack images shows that the entrapped cell number per unit height can be correlated with the formation of disulfide bonds and the PEG network. (C) As indicated by the images from the bottom of the ITO electrode, the crosslinking levels reached a maximum near 20 µm, as did the cell number. The low fluorescence in the upper regions of the hydrogel indicates a limitation of oxidized Fc in the upper regions, suggesting multiple oxidation steps may be needed or perhaps additional Fc (to provide more oxidation). *Data are presented as mean ± SD (*n* = 4).

We examined the Z‐stack (Figure 4A) data further and found a consistent bell‐like symmetric distribution along the vertical axis as the gels were formed. All profiles nearest the electrode were repeatedly consistent across all tested durations. Trajectories all suggest that early on, cells are deposited rapidly at the electrode, and as time progresses, the number of cells entrapped continues to increase, but mainly in regions where the new gel is formed and this interface continues to extend upward [32]. That is, once gels are formed, there appeared to be no additional cell addition at the regions having already been established. As noted earlier, the deposition of cells and gel is a consequence of Fc_oxi_ concentration, diffusion, reaction kinetics, and applied charge. That there appears to be a maximum of cells in the middle of a vertical plane, is somewhat unexpected in that one might suspect that the number of cells would be highest at the electrode and decrease in number with distance from the electrode.

Mechanics of settlement aside, to confirm the geometries of cells and gel in the vertical direction, we switched cells to those fluorescing red (a dTomato construct) and used a green fluorescent dye attached to the PEG. Specifically, we doped 50 µg/mL of FITC‐labeled thiolated‐PEG (0.1%) into the deposition solution and applied oxidative potential for 180 s. We replaced the DH5α‐sfGFP cells with DH5α‐dTomato cells which constitutively express red fluorescent proteins, enabling better imaging quality and contrast for the two entities. As before, we captured Z‐stack images with 5 μm resolution (cell number and gel green fluorescence profiles shown in Figure 4B and confocal images shown in Figure 4C). As the green fluorescence increased (indicating the PEG polymerization), so did the cell count, as expected. Moreover, there was close correspondence between the two entities. Our observations are consistent with established theoretical frameworks for electrode‐actuated gelation, which describe how spatiotemporal gradients in electrochemical signals dictate hydrogel formation [33, 35]. In previous work, we found a similar bell‐like profile in the same hydrogel and with cells. There, we used in situ Brillouin spectroscopy and found the gel's mechanical properties (crosslinking density) were also distributed in a similar fashion [32]. Together, these results indicate that (i) the formation of PEG polymer is closely associated with the cell entrapment, (ii) gel formation extends from the electrode surface and the process is consistent over time, such that (iii) once cells are trapped, there appears to be little additional entrapment, suggesting that the density of cells within a volume of gel increases in time as the gel front vertically moves from the surface, until it reaches a maximum of around 10^4^ cells/5 μm. We can suggest that as the gel crosslinks from the electrode surface outward, it entraps cells based on its crosslinking density.

### 2.3 Assembly Depends on Deposition Time, Mediator Concentration, and Cell Number Density

**FIGURE 5:**
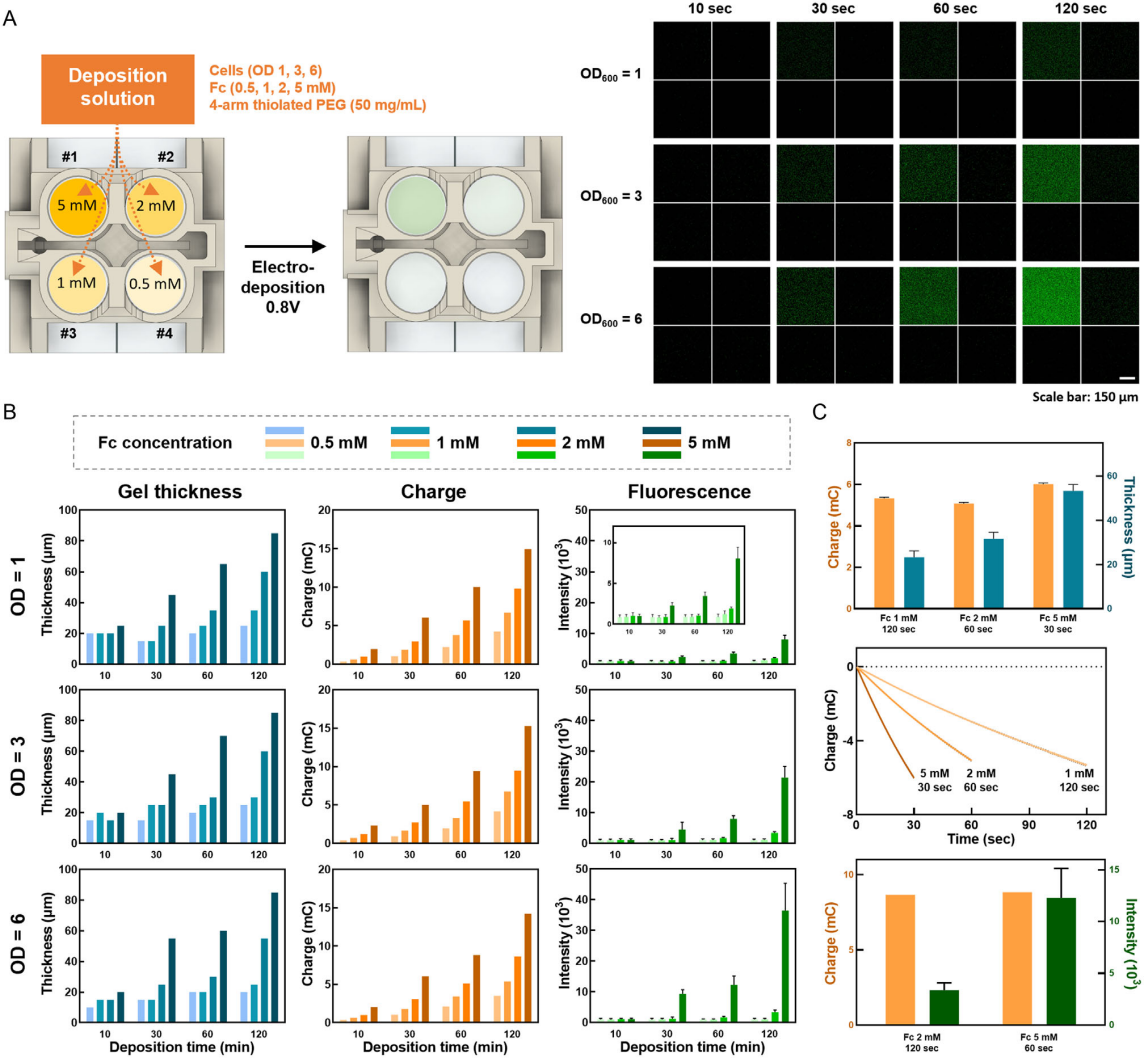
Hydrogel assembly as function of initial cell loading, mediator concentration, and applied potential. (A) Various Fc concentrations (0.5, 1, 2, and 5 mM), cell densities (OD_600_ = 1, 3, and 6) were tested with different durations of applied potential (10, 30, 60, and 120 s). Fluorescence (top‐view) images acquired by confocal microscopy enable the visualization of the entrapped cells in the gels. Strong fluorescence was found principally in tests with 5 mM of Fc. Weak fluorescence can be observed at 2 mM of Fc for longer deposition times; insignificant signals were obtained for lower concentrations and at 10 s deposition. (B) Charge was calculated as the accumulation of current over time. Gel thickness was highly correlated to the applied charge across all groups. Fluorescence was most significant in all 5 mM Fc cases. (C) Charge, gel thickness, and cell entrapment appear regulated by the mediator concentration. Three conditions resulted in comparable charge values (5.39, 5.138, and 6.08 mC, from left to right) but the thickness showed a significant increase only when mediator concentration was higher than 2 mM. In the lower panel, at both longer times and higher Fc concentrations, we found comparable charges (8.649 and 8.832 mC, respectively) but again, the fluorescence (indicating cells) here was high only at the 5 mM Fc case. The data was adopted from (B) *Data are presented as mean ± SD (*n* = 4).

The data in Figure 4 illustrate cell/gel assembly using the same initial cell density and Fc concentrations. Next, we systematically investigated how mediator concentration and cell density influenced the assembly process. Solutions with Fc (0.5, 1, 2, and 5 mM) were added to 50 mg/mL of PEG deposition solution also containing DH5α‐sfGFP cells with the following cell concentrations: OD_600_ = 1, 3, and 6. The solutions were then loaded into the wells of the ITO device followed by electrodeposition (+0.8 V) for different durations (10, 30, 60, and 120 s). We then removed uncured deposition solution, rinsed the wells three times with 200 µL of PBS, and examined gels using confocal microscopy (Figure 5). The gel height was defined by the last Z‐stack slice in which cells were first clearly visible. As above, we overlayed all Z‐stack images into one top‐view image to illustrate the overall fluorescence level (right panel of Figure 5A). Representative charge, thickness, and fluorescence intensity plots are shown in Figure 5B.

As the duration of deposition and the concentration of Fc increased, the calculated accumulated charge increased, as anticipated. Meanwhile, the correlation between charge and the PEG‐gel thickness was most prevalent with Fc concentration over 2 mM. That is, at low Fc concentrations, while there was clearly a monotonic increase in charge with increased Fc over time, there was no corresponding increase in gel thickness. We suggest that in cases where the gel thickness remained low, the incremental charge was likely due to redox cycling via redox reactions with components other than the free thiols on the 4‐arm PEG monomers (i.e., cell‐associated). That is, we note that charge was not found during similar studies but without cells [32], reinforcing cell‐associated redox activity. To verify this hypothesis, we observed that replacing cells with polymer microspheres allowed gel formation at significantly lower Fc concentrations (0.5 mM), suggesting that the presence of cells likely compromises a portion of the Fc oxidation power required for PEG crosslinking. Despite this, the maximum gel thickness remained limited to several hundred µm. As the redox mediator Fc serves as the electronic messenger to free thiols, this growth ceiling likely reflects diminished electron renewal at the electrode as mass transport resistance increases within the thickening gel matrix. Then, from data in Figure 5B, it would appear that success in cell entrapment was most prevalent in solutions with above 2 mM Fc, irrespective of the cell density. Correlations between charge and gel thickness measured based on the cell distribution are also shown in Figure S4.

Interestingly, we found that the rate of applied charge correlated to the extent of crosslinking and gel formation. That is, while the total charge applied for the 1 mM Fc case was nearly identical to that of the 2 and 5 mM cases (Figure 5C upper), the rate of charge transfer was dramatically lower (Figure 5C center). Correspondingly, the greatest thickness and number of the entrapped cells (i.e., gel polymerization) occurred at the highest Fc concentration or at conditions of the fastest charge transfer (Figure 5C bottom). These results suggest that the “energy density,” or redox cycling efficiency, governed by the mediator (Fc) concentration, played a decisive role in controlling gel formation and cell entrapment.

### 2.4 External Forces (i.e., Gravity) Influences Assembled Cell Density

**FIGURE 6:**
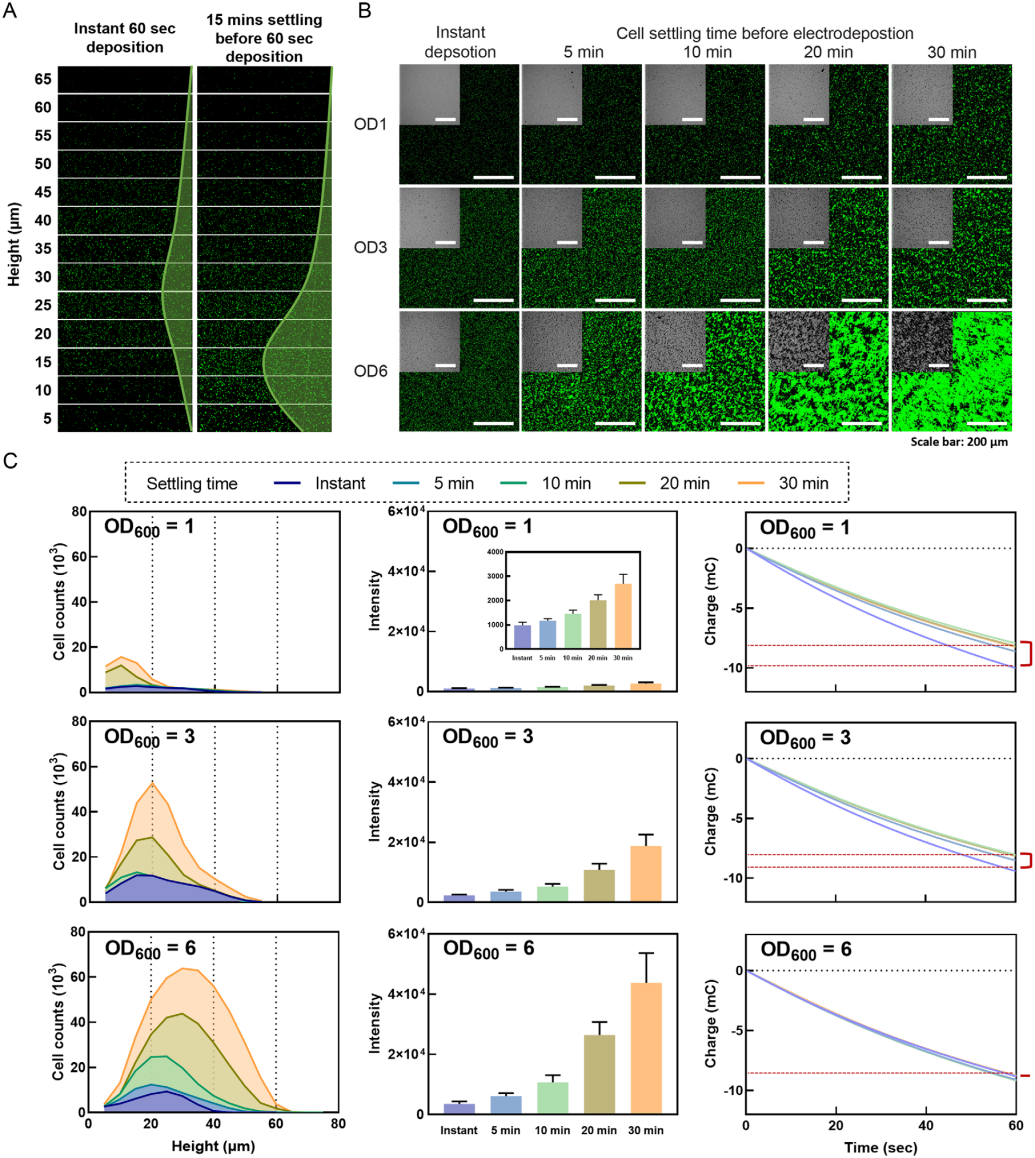
Dynamic manipulation of cell distribution in cell/gel assemblies via gravity settling. (A) Side‐by‐side comparison of confocal images for the two tested conditions. Left: Immediate application of oxidative potential for 60 s after providing the deposition solution to the well; Right: the deposition solution was allowed to settle for 15 min and then the identical potential was applied for 60 s. Both gels were found to be the same height, indicating that layered cell content did not influence gel formation. However, a layer of cells towards the vertical middle with significantly higher density was visible. (B) Bright field and fluorescence top‐view images obtained by confocal microscopy show that the accumulation and aggregation of cells contribute to the increased cell content in the defined volume. The formation of small clumps or branch‐like features by aggregated cells was dependent on initial cell density. (C) Cell distribution plotted from confocal Z‐stack images reveals that cell accumulation is a time‐dependent dynamic process. Cells tend to localize at the bottom when initial cell density is low, with the peak location similar across all settling times. In contrast, cells stack quickly and form a bell‐like distribution with the peak shifting vertically from the electrode when the initial cell density is high. This is consistent with hindered settling. The overall fluorescence intensity quantified using a plate reader demonstrates that gravity‐facilitated settling can significantly enhance the optical signal emitted from the entrapped cells due to increased cell density. *Data are presented as mean ± SD (*n* = 4).

In the sections above, we demonstrated that the initial cell density in the deposition solution was an important factor in establishing the final assembled cell density, especially for cases where the deposition duration was above 30 s and the Fc levels were 2 mM and above. Typically, the deposited cell gel networks were consistent across the tested parameter ranges—a vertical assembly with a maximum in cell density midway in the vertical direction. Also, while not noted, the maximum heights were typically ≈60 μm and rarely exceeding 100 μm from the electrode surface. We further sought to explore alternative, but simple, means to provide additional vertical features in the assembled structures. In previous work, we used magnetic nanoparticles and magnetic fields to provide additional structure for biomaterials assembly [36, 37]. Here, we exploit the simplest of external forces, gravity, as an additional tool for developing structure (i.e., stratified films). We examined two different settlement conditions in which cells were entrapped into a gel via PEG crosslinking either immediately after loading or waiting for 15 min prior to applying potential. In Figure 6A, we depict the Z‐stack images reflecting the vertical cell density after applying + 0.8V potential and 5 mM Fc at time zero (left, denoted “instant”) and after applying potential after 15 min during which time cells (initially OD_600_ = 1) could settle (right). Interestingly, in both cases, the cell densities at the uppermost levels remained comparable. Deposition solutions with three different initial cell densities (OD_600_ = 1, 3, and 6) were then tested. 100 µL of the samples were loaded and allowed to settle at room temperature for various durations (5, 10, 20, and 30 min). After the indicated times, an oxidative potential was applied for 60 s (Figure 6B); the fluorescence signal significantly increased across all conditions due to the increased number of entrapped cells. Similar results were observed across all tested conditions. We also noted there was appreciable agglomeration throughout (indicated by larger clumps, especially at OD_600_ = 6). In Figure 6C, we again used both a plate reader and confocal microscope to measure the overall fluorescence intensity and generated Z‐stack images for vertical distributions, respectively. While not shown here, the final gel thickness was ≈60 µm in all cases. As expected, the more initial cell loading, the more final cell density was immobilized. Also, the charge distribution was quite consistent between the cases, which aligns with each case having the same density of available PEG thiols for crosslinking within the 60 µm volume.

Under quiescent conditions, cells should settle and form a clear boundary between the clarified liquid above and the remaining suspension below; and this boundary should move downward in time. According to Kynch's flux theory [38], this settling front remains sharp and moves in accordance with particle concentration in the initial suspension; classical hindered‐settling behaviors explain how higher cell densities slow this motion [39]. In non‐Brownian suspensions such as those here (i.e., with large particle sizes) higher cell concentrations should slow the front. In our system, this means that more concentrated cell suspensions that are well‐mixed initially should produce a slower settling front, initiating at the highest vertical positions. By contrast, lower cell density suspensions, in particular those that present less impedance to cell deposition, should enable a more rapid settling that is more aligned with the classical Stokes settling velocity that assumes no particles in the vicinity. Interestingly, in the left panel of Figure 6C, we observed a peak in cell number along the vertical direction that was lower for the low‐cell‐density conditions (initially OD_600_ = 1) than for the higher cell density conditions (initially OD_600_ = 6). This is in support of a cell density‐dependent hindered settling hypothesis. Our charge data obtained for the 60 s over which the oxidative potential was applied (right panel, Figure 6C) can also provide insight regarding this settling process. First, when there was no settling (denoted “instant”, as above) and the potential was applied directly upon sample addition to the device, the charge profile showed a rapid monotonic decrease to ≈−9 mC. There were no obvious differences between the 1, 3, and 6 OD_600_ levels, suggesting that for the OD_600_ levels tested, there was no influence of cell density on charge for the initial 60 s. Then too, at 6 OD_600_ (which would present the most hindered settling condition), there was very little difference between the “instant” settling and all the other settling times. This could suggest that at OD_600_ = 6, there was no significant settling front established during the 60 s of applied charge that was also detected at the electrode, nearly 60 μm away. In other words, the cell suspension at the initial state remained steady near the electrode. The data also reveals a cell density dependent charge for the other initial OD_600_ cases. That is, samples subjected to cell settling exhibited lower charge outputs compared with the control (“instant”) case. A reduction in charge was observed, and this was more pronounced under low‐cell‐density conditions (initial OD_600_ = 1). We attribute these observations to the accumulation of cells on or near the electrode surface, which likely impeded the transport and diffusion of the redox mediator. The lowest cell density cases that settled for the longest time, had more cells in the vicinity of the electrode. Consistent vertical distribution profiles were observed when the bacteria were replaced with fluorescent micro‐beads of a similar size, further validating the physical sedimentation and entrapment mechanism. However, unlike the cellular groups, no significant aggregation was found in the micro‐bead control groups. This suggests that the clusters observed in the cell‐laden gels are primarily driven by intercellular forces and biological interactions (such as cell–cell adhesion or EPS‐mediated bundling) rather than purely stochastic physical collisions during the electrodeposition process (Figure S5).

Finally, we point out to that the cell numbers quantified by the Z‐stacked images are quite consistent with the fluorescence measurements taken by the plate reader (Figure S6). After 30 min of settling, the fluorescence signal increased by 175.31%, 716.86%, and 1132.45%, respectively, across tested initial cell OD_600_ (1, 3, and 6). Thus, by enabling cell settling, one could assemble more total cells into the gel matrix, and the increase was proportional to the increased settling time and initial cell number. Since the total height was equivalent among the varied cases, we suggest that the number of cells assembled into these matrices has not yet been maximized.

### 2.5 Electrochemical “Programing” to Provide Structure

**FIGURE 7:**
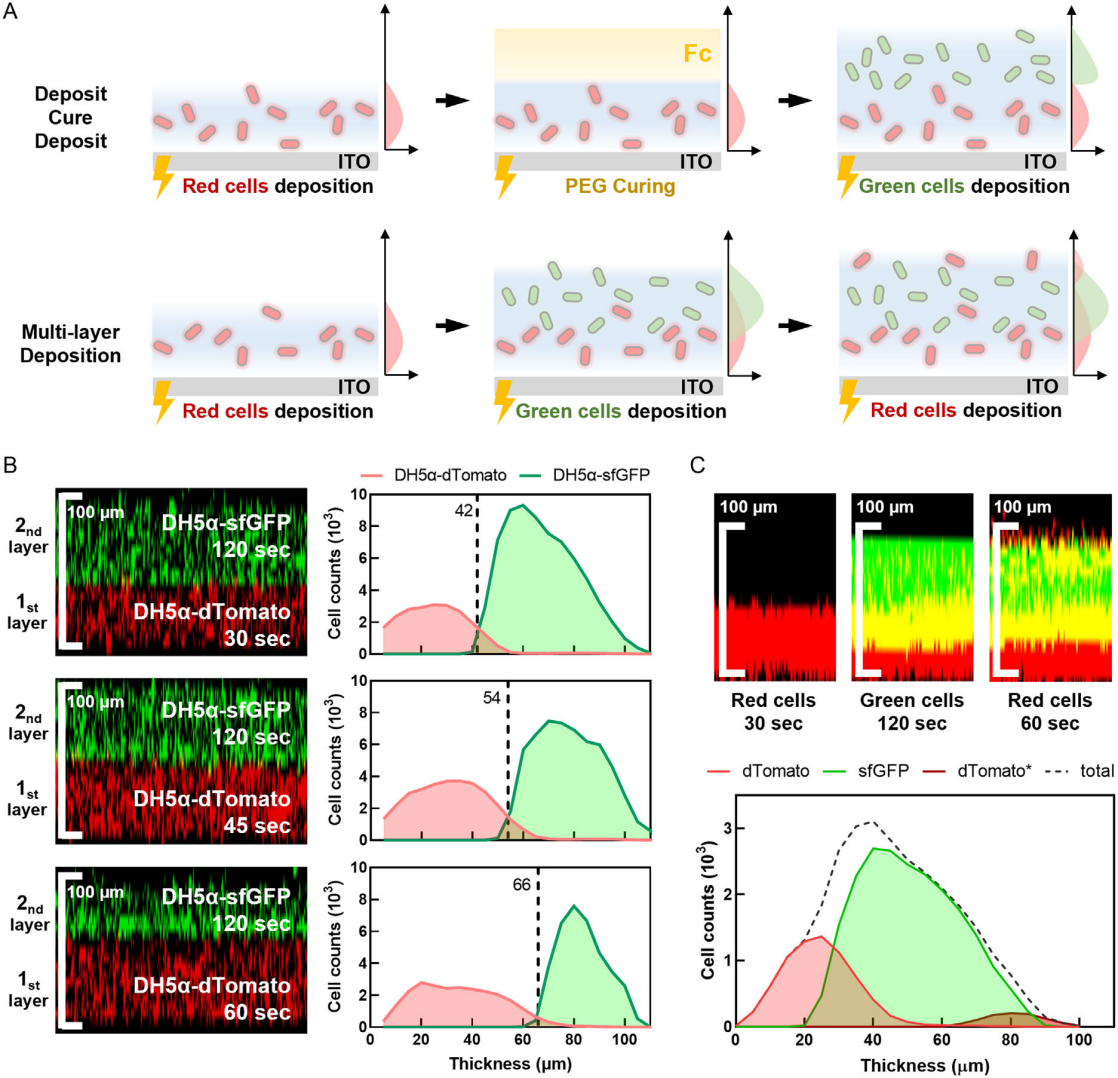
Controllable artificial biofilm boundaries. (A) The upper scheme demonstrates a “Deposit‐Cure‐Deposit” method for “closing” the surface for subsequent assembly. The bottom scheme shows steps for multi‐layer cell/gel formation. (B) Mediated with the Fc, an additional oxidative potential was applied to the hydrogel after the first deposition, seemingly electrochemically facilitating the crosslinking of free thiol groups and suppressing the entrapment of more cells during an additional deposition process. Note that the deposition time for the first layer determines the position of the boundary between two cells. (C) Results indicated that simple addition of layered gels (without “curing”) enables comingled entrapment of cells within the next lowest layer.

Because (1) the applied charge serves to quickly crosslink the gels, (2) the vertical cell distribution profiles was typically bell‐like, and (3) one can use gravity to create layers, we decided to see if we could alter the apparent porosity between layers and, in effect, create layers that had either well‐defined distinct boundaries or that were more porous enabling cells of two vertical populations to mix at the interface. This is shown schematically in Figure 7A (upper panel). As shown in Figure 4, we hypothesized that the upper region of the bell‐shaped profiles (where cell counts decline) corresponds to areas containing “uncured” or “un‐crosslinked” thiolated PEG, owing largely to the distance from the electrode. In principle, this region's cell‐entrapping capability could be selectively tuned by regulating the availability of free thiol groups. Here, we wanted to see if we could “seal” or “cure” the gels using additional oxidative thiol crosslinking and whether this would serve to sharpen the interface between cell layers. That is, we suspected that continued charge for crosslinked thiolated PEG could continually oxidize thiols as long as Fc cycling occurred. Specifically, we added 100 µL of deposition solution containing DH5α‐dTomato cells (OD_600_ = 6) that constitutively express red fluorescent protein, thiolated‐PEG (50 mg/mL), and redox mediator Fc (5 mM) into the wells of the ITO devices. Following the previously established protocol, an oxidative potential of +0.8 V was applied to the ITO electrode to form the PEG gels. As previously shown, we constructed this first cell layer for various deposition durations (30, 45, and 60 s) and washed the wells with PBS to remove residue. After washing, we added PBS containing a fresh 5 mM of Fc and attempted to electrochemically “cure” the first layer by applying oxidation for an additional 30 s. We then washed and using the same protocol as above, assembled a second layer of DH5α‐sfGFP cells in PEG solutions (green fluorescent, OD_600_ = 6) by applying the 0.8V but for a longer time, 120 s. Confocal images were taken after each wash step. We then merged the Z‐stack images at the resolution of 5 µm. To better visualize where the images of red and green are combined, we denoted these regions in yellow. The results suggest there is only very limited vertical region that was occupied by both cell types (i.e., little overlap) (Figure 7B).

In the next set of experiments, we carried out the identical process, but instead of a second applied potential with Fc, we deposited the second layer of cells without the curing process (Bottom panel of Figure 7A). Following the previously established protocol, we constructed the first cell layer for 30 s and washed the wells to remove residue and filled the wells with only PBS. However, in this case, the additional charge was applied to an environment that had no additional Fc, so there was likely no additional crosslinking and instead of a “sealed” layer, the boundary could hypothetically be a bit more porous. We then removed the liquid and refilled the well with 100 µL of the deposition solution containing DH5α‐sfGFP cells (OD_600_ = 6) that constitutively express a green fluorescent protein, thiolated‐PEG (50 mg/mL), and redox mediator Fc (5 mM) and then generated the second cell layer using +0.8 V for 120 s (Figure 7C, upper middle). In this case, the yellow band (indicating both green and red cells) was somewhat thicker than the red band of the left image. To build further, we then repeated the same protocol with a PBS‐only charge after the second layer for subsequent assembly of a third cell layer with red fluorescent DH5α‐dTomato cells for 60 s, again without “sealing”. In this case, there was a larger yellow band, as illustrated in Figure 7C (upper right). Careful distribution analysis of the layers reveals three bell‐like profiles in the vertical direction from the bottom ITO electrode, with the second layer containing green fluorescent cells contributing the majority of the cell content (Figure 7C). The yellow swath in this image suggests that some of the red cells added in the last layer were indeed incorporated into the network. Note again that the initial OD of each layer was identical in this set of experiments.

Interestingly, in the earlier studies also using 100 μL deposition solutions with varied cell number at maximum of 5 mM Fc with charge applied for 60 s, we often found gels of ≈60 μm. The total thickness for the two‐ and three‐layered approaches, here, was typically ≈100 µm. While one might expect the total thickness to reflect the total available free thiols, we found just a thin layer of red fluorescent cells at the top region of the second layer, suggesting other limitations to the total height. That is, while the deposition was nearly always uniform in 2D, the distribution of cells in the vertical position was a function of many variables including the number of cells, the concentrations of Fc and PEG, and whether or not one used settling or “curing” to help define vertical geometries. That said, without settling or “curing”, the gels made for entrapping a population of cells were highly regular and proceeded smoothly with time.

### 2.6 Electroassembly of Multiple Cell Types Onto Conductive Materials of Varied Geometry

**FIGURE 8:**
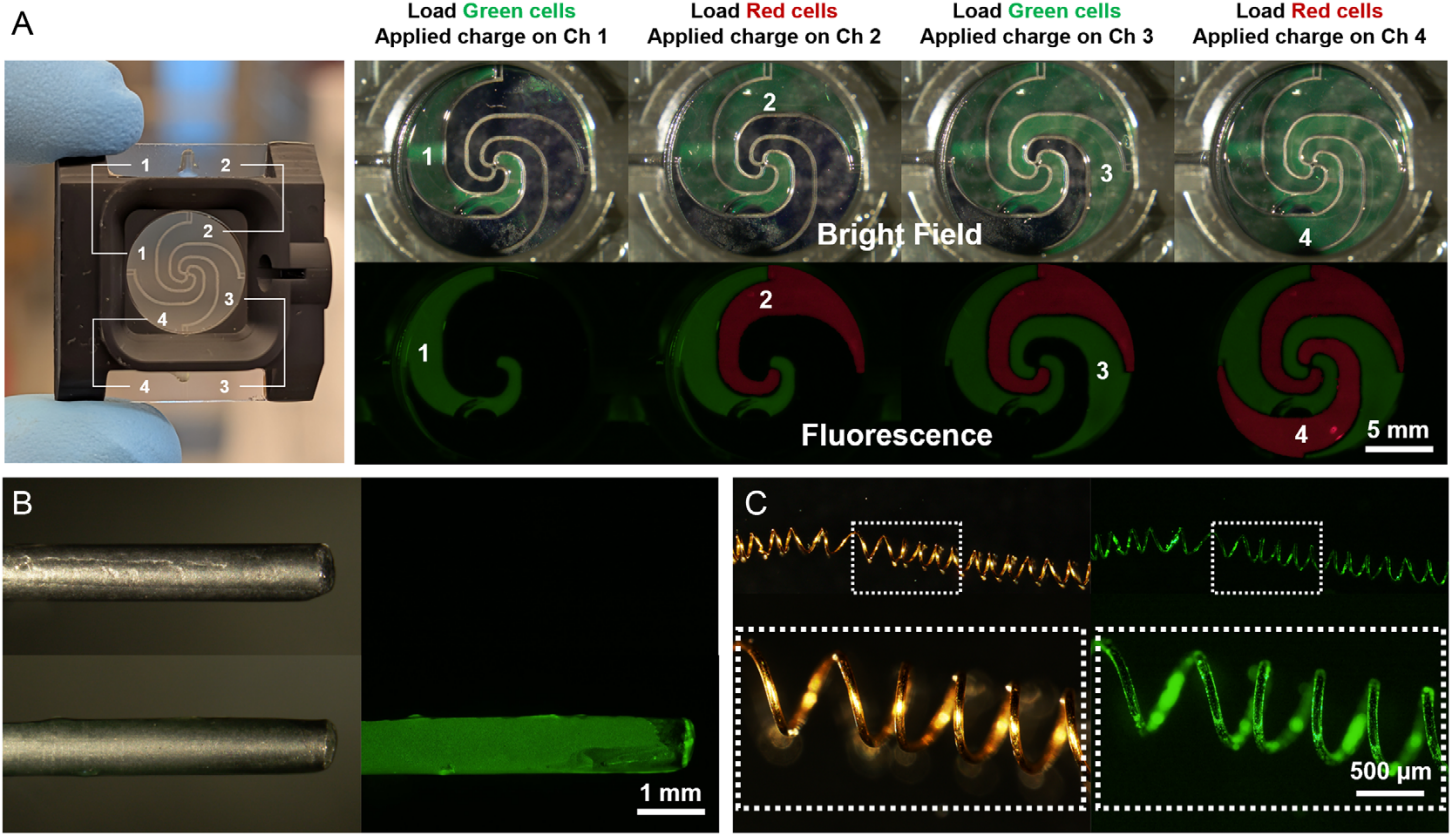
Location‐specific electroassembly onto various electrode materials. (A) Patterned ITO electrode designed within a single 3D‐printed well with lollipop shaped electrodes, enabling location‐specific PEG gel formation and cell entrapment without cross‐contamination between electrodes. (B) PEG hydrogel polymerized on a pencil lead, showing uniform coating (top and bottom) and fluorescence when DH5α‐sfGFP cells were included (bottom). (C) Gold coil electrode (50 µm wire) after electroassembly at +0.8 V for 60 s; a thin PEG layer is visible (left) and with fluorescent cells on the surface (right).

The previous results were prepared using working electrodes at the bottom of 3D‐printed wells; all 4 electrodes shared identically configured liquid volumes. Because electroassembly is enabled by the electrode properties (including their geometry [17, 29, 38, 39, 40, 41]), we wanted to test whether the thiolated PEG process was sufficiently generic so that cells could be “programed” for assembly onto a variety of shapes and using a variety of conductive materials. In Figure 8A, we patterned a series of ITO electrodes with an intertwined (lollipop) pattern. Following the previous protocol, we loaded the deposition solution containing DH5α‐sfGFP cells (OD_600_ = 6) and applied + 0.8 V for 60 s onto the first electrode, then thoroughly rinsed the with 1 mL of PBS three times. Next, using the same procedure, we crosslinked gels on the 2nd, 3rd, and 4th electrodes with deposition solutions containing DH5α‐dTomato cells (OD_600_ = 6), DH5α‐sfGFP cells (OD_600_ = 6), and again DH5α‐dTomato cells (OD_600_ = 6), respectively. Bright field and fluorescence imaging confirmed that the gels containing target cells were successfully assembled onto each subsequent electrode and, remarkably, there was no apparent cross contamination. That is, in our previous studies, we observed that electrode‐generated pH gradients used to create chitosan or alginate gels could result in crosstalk owing to the non‐specific binding properties of the biopolymers [42, 43]. In contrast, here, there was no apparent cross‐contamination using PEG and ITO. Next, as shown in Figure 8B,C, we applied this method to various conductive materials, including a graphite rod (i.e., pencil lead) and a hand fabricated coil made from 50 µm gold wire. We polymerized PEG hydrogels both with and without DH5α‐sfGFP cells on the graphite rod. Brightfield images confirmed uniform PEG coating on both rods, while fluorescence was observed only on the rod containing cells. Using the same deposition solution and protocol, we further applied +0.8 V to the gold coil for 60 s, resulting in a visible thin gel layer on the surface with clearly identifiable green fluorescent cells under the fluorescence microscope. Finally, to evaluate the capability of the proposed protocol for multiple cell types, we have electroassembled CHO cells, yeast, algae, and a different bacterium (*Pseudomonas*), and confirmed the cell viability and various functionalities after the deposition process (Figures S7).

## 3 Conclusion

In this study, we “programed” the electroassembly of thiolated PEG hydrogels and the incorporation of bacterial cells onto electrodes of various geometries and materials. Our results show how the assembly was influenced by the Fc mediator concentration, initial cell number, the chosen oxidative potential, a self‐imposed settling time, and the application of “curing” steps. We showed that a variety of vertical patterns can be created, and, in all cases, cells retained their function (denoted by expressing proteins, consuming substrates, etc.) and viability. We found that a second extended “curing” oxidation step seemed to create a less porous gel surface that prevented the integration into the first layer of cells assembled in subsequent layers. In sum, we assert that the creation of cell layers onto optically clear substrates, wherein engineered cell functions can be maintained, enables a simple and reproducible way to study cell‐based systems, including those of different populations, and to decorate various devices of various geometries and materials with engineered living materials. We believe application of this work will find utility in health, energy, and environmental fields where the assembly of cells in many specified geometries enables their detailed study and potential application.

## 4 Materials and Methods

### 4.1 Materials

Indium tin oxide‐coated glass slide, square (surface resistivity 8–12 Ω/sq), and phosphate‐buffered saline (PBS, pH 7.4) were purchased from Sigma–Aldrich (St. Louis, MO). Black 3D printing resin was purchased from ELEGOO (Guangdong, China). 1,1′‐Ferrocenedimethanol (Fc) was purchased from Santa Cruz Biotechnology (Dallas, TX). A 1 M stock solution of Fc was prepared in DMSO, and dilutions were prepared in PBS freshly for each experiment.

### 4.2 Device Fabrication and Performance Check

We employed the design of our previously developed optically transparent indium tin oxide (ITO) 3D‐printed platform that enables concurrent electrochemical and optical measurements. In brief, the 3D‐printed device housing was assembled with the ITO glass with laser‐engraver electrode patterns to form the final test platform.

### 4.3 Cell Culture Protocol

*E. coli* reporter cells (DH5α‐sfGFP or DH5α‐dTomato) that can constitutively express high levels of sfGFP were cultured overnight in the Luria‐Bertani broth (LB) in a 37°C shaker, and the supernatant was removed (centrifuged at 3000 × g for 10–15  min) and the cells were washed twice with PBS and resuspended to the desired concentration in PBS before use. *Pseudomonas chlororaphis* was cultured (10 mL) in LB Lennox media (NaCl, 5 g/L, Tryptone, 10 g/L, Yeast Extract, 5 g/L) at 30°C, grown to an OD600 ≈1. Cells were centrifuged at 3000 × g for 10 min, washed once in PBS, and resuspended in 1 mL PBS. CHOZN23 (Millipore Sigma) cells were cultured in DMEM with 10% FBS in a 37°C incubator with 5% CO_2_ and harvested and resuspended to the desired concentration in PBS before use. Yeast (*Saccharomyces cerevisiae*) was cultured (10 mL) in Yeast Peptone Dextrose (YPD) broth (Difco, 242820) at 30°C, grown to an OD600 > 1. Cells were centrifuged at 3000 × g for 10 min, washed once in PBS and resuspended in 1 mL PBS. Algae (*Chlamydomonas reinhardtii*) were cultured in 1 L spinner flasks in Tris Acetate Phosphate (TAP) media [44] for 5–6 days at 25°C under ambient light. 10 mL culture was sampled from the 1 L culture, centrifuged at 3000 × g for 10 min, washed once in PBS, and resuspended in 1 mL PBS.

### 4.4 Electrodeposition of PEG‐SH and Immobilization of Cells

Electrodeposition was performed using the designed 3D‐printed 4‐well device with ITO electrodes and a docking station [10]. Voltages were applied, and charges were recorded using CHI1040C electrochemical analyzer (CH Instruments; Austin, TX). 2X stock solutions containing 100 mg/mL PEG‐SH with various Fc concentrations (1, 2, 4, and 10 mM) were prepared in 100 mM potassium phosphate buffer (pH = 7.4) and then mixed with 2X cell stock solutions prepared in PBS (OD_600_ = 2, 6, 12) to form the final cell/PEG‐SH/Fc solutions. 100 μL of the solutions were loaded into the wells and applied with constant voltage for varying durations (10, 20, 30, 45, 60, 120, and 180 s), unless otherwise noted. A constant voltage of +0.2, +0.5, +0.8, and +1.0 V was applied to induce PEG‐SH crosslinking for initial tests; +0.8 V was used for the remaining experiments. Unreacted gel deposition solutions were immediately removed from the wells after deposition, followed by 3 times of PBS rinse to remove residues. 100 μL of PBS was then added into the wells for the ensuring tests. Plate reader (TECAN Spark plate reader (Tecan Group Inc.; Männedorf, Switzerland)) and Zeiss Axio Observer Z1 confocal fluorescence microscope (Carl Zeiss AG, Jena, Germany) were then used for fluorescence measurement and cell counting.

For the FITC‐labeled thiolated‐PEG and DH5α‐dTomato cells codeposition tests: 100 μL of the deposition solution containing 50 µg/mL of FITC‐labeled thiolated‐PEG and DH5α‐dTomato cells (OD_600_ = 12) was added into the wells. An oxidative potential (+0.8 V) was applied for 180 s to electroassemble the hydrogel. Unreacted deposition solutions were immediately removed from the wells, followed by three sequential rinses with PBS to remove residues. 100 μL of PBS was then added into the wells for imaging. Same model of plate reader and confocal microscopy mentioned above were then used for fluorescence measurement and cell counts.

For the tests of biofilm boundaries: 100 μL of the deposition solution containing DH5α‐dTomato cells (OD_600_ = 2 or 12) was added into the wells. An oxidative potential (+0.8 V) was applied for varying durations (30, 45, and 60 s) to electroassemble the 1^st^ layer of hydrogel. The unreacted solutions were immediately removed from the wells followed by three sequential rinses with PBS to remove residues. 100 μL of PBS with or without 5 mM of Fc was then added to the well immersing the 1^st^ layer of hydrogel. An oxidative potential (+0.8 V) was repeatedly applied for 30 s. The deposition solutions were immediately removed from the wells followed by three sequential rinses with PBS. 100 μL of the deposition solution containing DH5α‐sfGFP cells (OD_600_ = 2 or 12) and 5 mM of Fc was added into the wells. An oxidative potential (+0.8 V) was applied for 120 s to form the 2^nd^ layer of hydrogel. The unreacted solutions were immediately removed from the wells followed by three sequential rinses with PBS to remove residues. 100 μL of PBS were then added into the wells for the coming tests.

The electrobiofabrication method was successfully extended to encapsulate mammalian cells (CHO), fungi (Saccharomyces cerevisiae), algae (Chlamydomonas reinhardtii), and bacteria (Pseudomonas chlororaphis) using standardized parameters (0.8 V potential, 5 mM Fc, and 50 mg/mL thiolated‐PEG).

### 4.5 Cell Imaging and Fluorescence Detection

All devices were examined using a Zeiss Axio Observer Z1 confocal fluorescence microscope (Carl Zeiss AG, Jena, Germany) for imaging and/or they were inserted into a TECAN Spark plate reader (Tecan Group Inc.; Männedorf, Switzerland) with a docking station [10] for fluorescence measurements. The images were processed using ImageJ and Lightroom (Adobe, Mountain View, CA).

## Funding

This work was supported by the Biological and Environmental Research (DE‐AC52‐07NA27344), Gordon and Betty Moore Foundation (11395), Division of Molecular and Cellular Biosciences (2227598) and Defense Threat Reduction Agency (HDTRA1‐19‐1‐0021).

## Supporting information

Supplementary Material

## Data Availability

All data, including images of hydrogels, is freely available upon request.
