# Supplementary material for: Programable and Spatially Conforming Assembly of Engineered Living Materials Onto Electrodes via Redox
Source: Small Sci. 2026 Jul 12;6(7):e70331. doi: 10.1002/smsc.70331 (PMC13387288; doi:10.1002/smsc.70331)
Supplement: Supplementary file 1 — Supplementary Material [file SMSC-6-e70331-s001.pdf]

Programmable and Spatially Conforming Assembly of Engineered Living Materials onto Electrodes via Redox

Chen-Yu Chen[1,2,3], Monica J. Chu[1,2,3], Fauziah Rahma Zakaria[1,2,3], Eunkyoung Kim[2,3], Divya Muthusamy[1,2,3], Gregory F. Payne[2,3*], William E. Bentley[1,2,3*]

[1]Fischell Department of Bioengineering, University of Maryland, College Park, Maryland, United States

[2]Institute for Bioscience and Biotechnology Research, University of Maryland, College Park, Maryland, United States

[3]Robert E. Fischell Institute for Biomedical Devices, University of Maryland, College Park, Maryland, United States

Correspondence:

William E. Bentley

Fischell Department of Bioengineering,

Institute for Bioscience and Biotechnology Research,

Robert E. Fischell Institute for Biomedical Devices,

University of Maryland, College Park, Maryland, 20742, United States.

Email: bentley@umd.edu

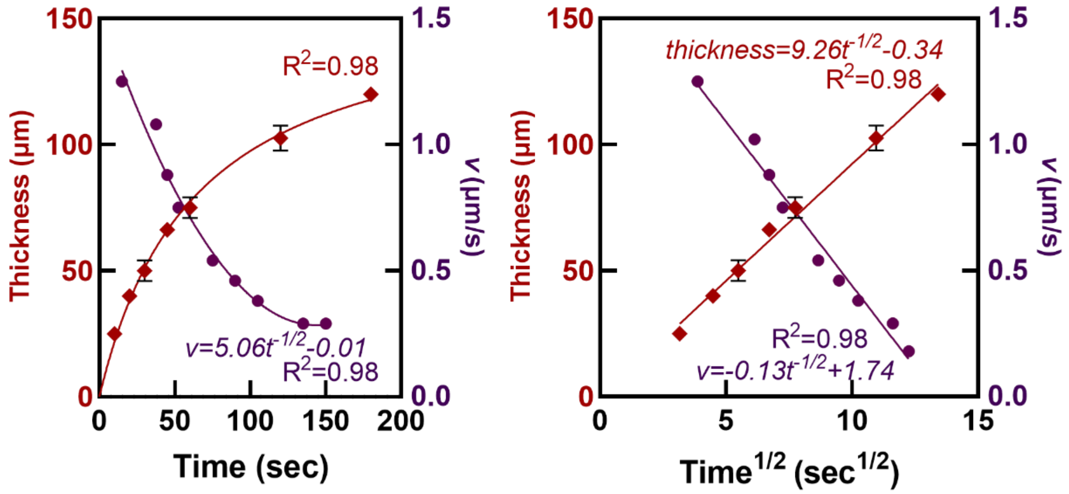

Figure S1. Diffusion-controlled redox-catalyzed gelation.

The reaction front, indicated by the gel thickness, advanced most rapidly during the initial 30 sec (approximately 1.2–1.3 m/s, not shown). The propagation speed then gradually decreased to about 0.5 μm/s after 60 sec and eventually approached 0.3 μm/s. This behavior is characteristic of a diffusion-controlled reaction front, where the velocity decreases proportionally to *t*^(−1/2).

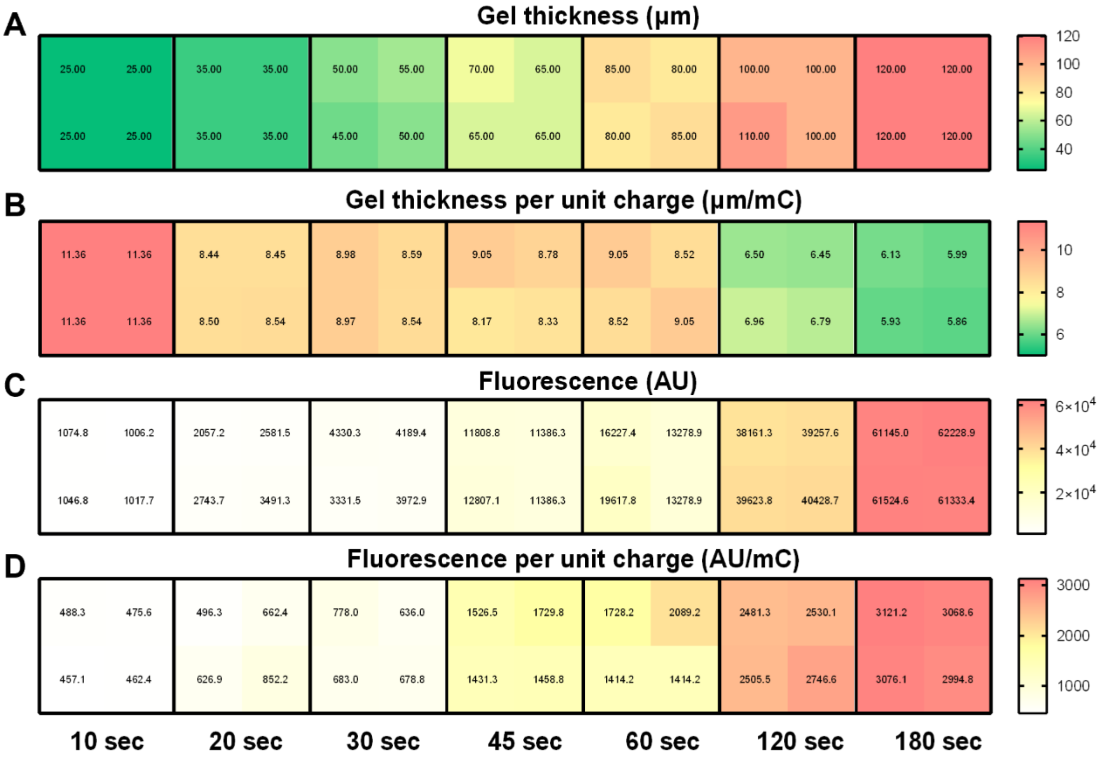

Figure S2. Time-dependent gel thickness and fluorescence.

Here, we used heat maps to demonstrate how deposition time impacts the assembled cell/gel features. (A) The gel thickness increase corresponds with the mediator-facilitated oxidation and the duration of applied potential. (B) The descending values of gel thickness per unit charge illustrate that later in the crosslinking process there is less oxidation of the thiols, suggesting that as the gel becomes thicker, there is increasingly less reaction overall so that the increase in gel height slows. (C-D) The total fluorescence increased slowly at first and then more rapidly after 60 seconds of applied charge.

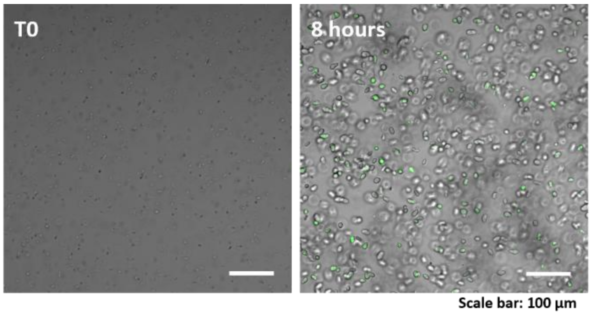

Figure S3. Cell retention test.

To evaluate cell retention, the assembled PEG gel with cells were subjected to three PBS washes and subsequently incubated in PBS for 3 hours. Minimal cell leakage was detected in the buffer, and no significant changes in gel integrity or properties were observed. Furthermore, when incubated in LB media for 8 hours at 37°C, the entrapped cells proliferated into localized, colony-like clusters within the hydrogel matrix. The absence of significant planktonic cell growth in the surrounding media confirms that the cells remained securely confined within the PEG network and were not released during incubation.

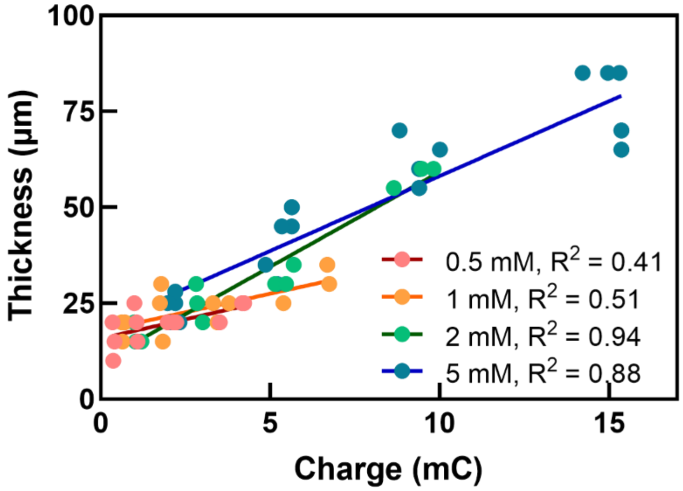

Figure S4. Correlations between charge and gel thickness measured as a function of mediator concentration and applied charge.

The results show that the concentration of the redox mediator, Fc, plays a role in the electrochemical crosslinking process. Gel formation occurred only when the Fc concentration exceeded 2 mM.

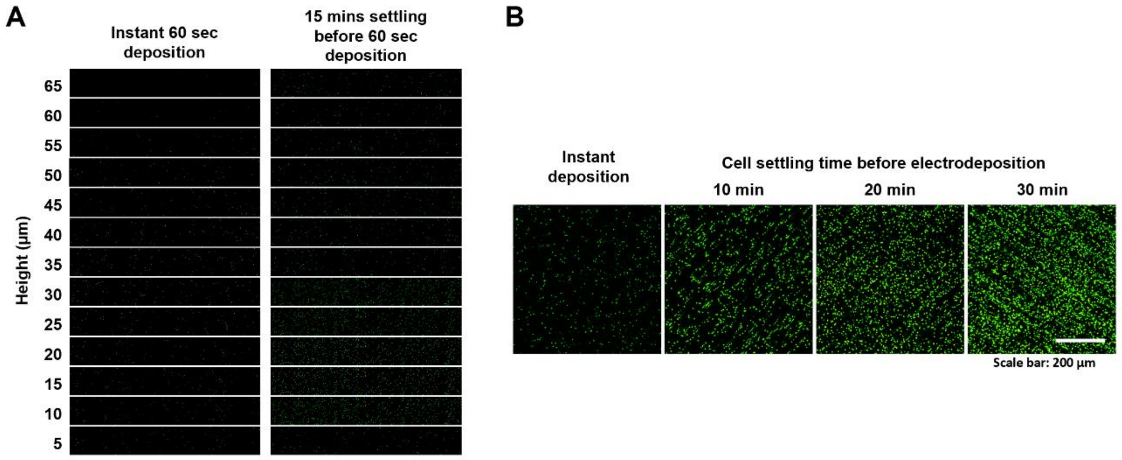

Figure S5. Vertical distribution of synthetic microspheres.

Control experiments using fluorescent polystyrene microspheres (~2 µm) were performed to decouple physical dynamics from biological activity. The resulting vertical profile of the microspheres closely matches the bell-shaped distribution observed in cell-laden gels, suggesting that the distribution is primarily governed by the interplay between sedimentation and entrapment kinetics. Beyond passive settling, the vertical heterogeneity likely reflects variations in crosslinking density and hydration levels during the gelation process. This is consistent with previous *in situ* Brillouin spectroscopy studies[30], which reported similar bell-like profiles for the longitudinal storage modulus of cell-embedded hydrogels, indicating a strong correlation between matrix mechanical properties and entrapment efficacy.

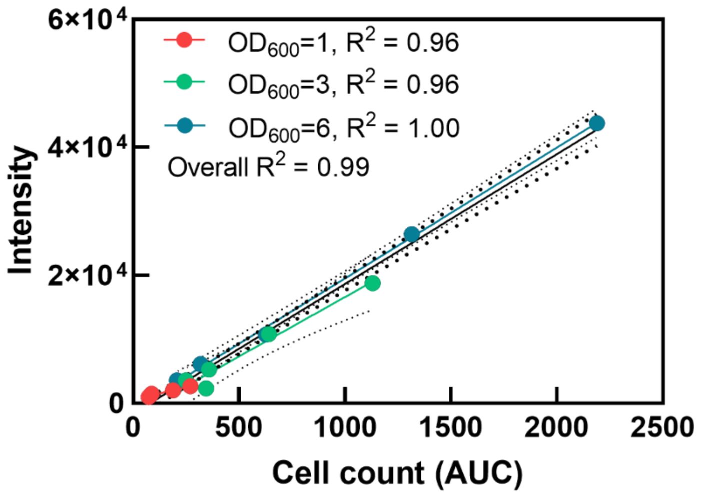

Figure S6. Correlations between fluorescence intensity measured from plate reader and cell counts in the artificial biofilm.

The intensity can also be correlated with the area under the curve (AUC, representing total number of entrapped cells). The plot illustrates a strong correlation between these two factors regardless of the initial cell density. The black line denotes a linear regression over all data points ($R^2 = 0.99$).

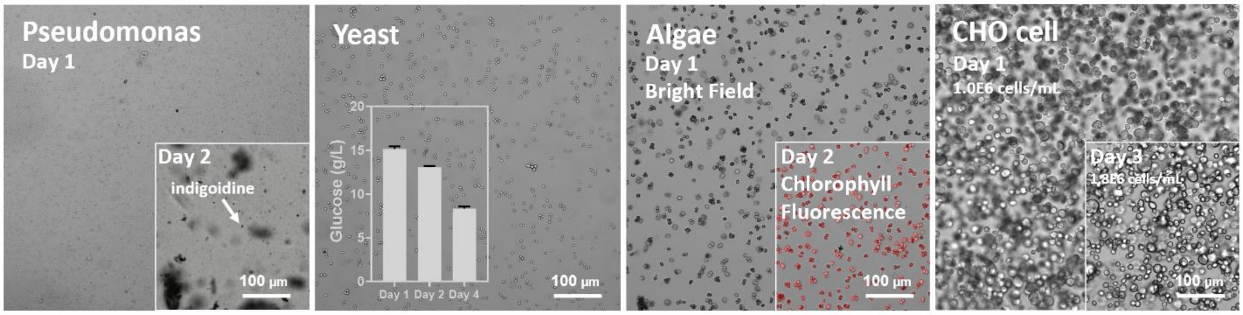

Figure S7. Platform versatility across diverse biological kingdoms.

The electrobiofabrication method was successfully extended to encapsulate mammalian cells (CHO), fungi (*Saccharomyces cerevisiae*), algae (*Chlamydomonas reinhardtii*), and bacteria (*Pseudomonas chlororaphis*) using standardized parameters (0.8 V potential, 5 mM Fc, and 50 mg/mL thiolated-PEG). For CHO cell, we monitored cell density for a subserquent 3 days and recorded the cell proliferation; for algae, we confirmed its strong chlorophyll fluorescence after culturing in the PEG gel for 2 days; for yeast, we monitored glucose consumption and recorded a decrease in glucose level in the medium after 2 days; finally, we cultured engineered *Pseudomonas* that in metabolically active cells produce indigoidine. We recorded the corresponding blue precipitation on Day 2.
